# Maternity and Mothering

**Published:** 1911-11-18

**Authors:** 


### MATERNITY AND MOTHERING.

(Inquiries and Correspondence are invited.)

### Rest and Exercise after Confinement.—I.

In different countries and in different classes the widest diversity of custom and practice prevails as to the length of time which is spent at rest in bed by the new-made mother after her confinement. This word confinement itself shows that, when our language was in the making, the need for some such restriction of activity during the puerperium was recognised in this country; but exactly how long that restriction ought to last has been a matter of great latitude. Recently it has been held by several eminent and ingenious obstetricians, chiefly on the Continent, that our usual customs err on the side of excess of rest, and that the health of mother and child are better promoted by an earlier return to ordinary habits.

Among savage tribes, as is well known, lying-in is practically unknown. The process of labour is usually quick and easy, and is not allowed to interfere with the usual routine of a woman's daily work. Amongst civilised peoples the conditions are, of course, so much altered that this happy-go-lucky nonchalance is impossible. Labour is a much more prolonged and much more exhausting affair; chiefly, it is believed, because the heads of white children are very much larger at birth than those of the black races. But it is interesting to recall in this connection an old custom, now fast dying out, which persisted up to a generation or two ago in the Yorkshire dales, and probably in other remote and sequestered parts of the kingdom. It was considered creditable, among the farmer population, for a mother to arise at once after the conclusion of childbirth, and do her ordinary work about the farm for the day; such as feeding the fowls and pigs, milking the cows, superintending the dairy, and so on. Having done this, presumably to demonstrate her courage or her robustness, the mother would retire to bed and spend a week or ten days there in the way usual amongst the urban population. The system was responsible for quite a lot of ill-health and even disasters, and its abandonment is doubtless due to the spread of more hygienic customs through the efforts of the medical profession.

At the present time the custom which is almost universally followed in Great Britain by the medical profession is to regard ten days as the minimum ordinary stay in bed for uncomplicated cases of normal puerperium in patients of average health and strength. Amongst the labouring class it is not uncommon for a shorter period to be regarded as sufficient; and amongst the well-to-do and somewhat pampered classes a slightly longer indulgence is often claimed successfully. At the lying-in hospitals, for example, the vast majority of the patients leave on the tenth day after their delivery; and, of course, this entails getting up two or three days previously.

In addition to this there are wide variations between the practices of obstetric physicians with regard to such questions as sitting, or being propped up, in bed; the strictness or otherwise with which the dorsal recumbent posture is enforced, and on similar questions. Some, for instance, allow the recently delivered patient to turn over or to kneel for the purpose of micturition within a few hours of childbirth; others will not even allow a pillow for the head for the first twenty-four hours. Some insist that none of their patients shall rise from bed until the lochia have entirely ceased; others view with indifference anything beyond an excessive duration of the lochia rubra, so long as involution appears to be proceeding normally and there is no other contra-indication of a resumption of ordinary habits of life. As a rule no exercises or exertions of any kind are suggested or allowed during this period, and muscular action of any kind is at a minimum. The reason for this is the fear of thrombosis and embolism, owing to the extensive uterine veins and sinuses whose lumina are undergoing retraction and obliteration.

Of late years, as has been hinted, German and other physicians abroad have been striking out new lines of practice in connection with these very important matters. These will be briefly considered in a subsequent article; it will be sufficient here to say that, notwithstanding the enthusiasm with which they preach the doctrine of early rising for puerperal women, they have quite failed to convince both the mass of the profession in this country and those specialists who have taken courage to put these novel theories from abroad to the test of actual practice.

At the same time it may be admitted that our British methods are sometimes too inelastic; or, rather, that they are interpreted without the necessary intelligence and originality which every doctor should possess. To endeavour to adapt patients to rules is as foolish as to try to fit facts to theories. The study of the individuality of patients, be they men, women, or children, is essential to successful practice, and nowhere is this important truth better illustrated than in relation to the matters now under consideration. No two women are endowed with quite the same original powers of endurance, of recuperation, and of resistance or response to environment; and, if it can hardly be said that no two labours are exactly and precisely the same, still there are at least very wide differences between them. The physician who studies his cases carefully soon learns the justice of all this, and abandons any arbitrary standard to which all his patients are to be made to conform. The state of involution of the uterus; the colour, quantity, and other characters of the lochia; the pulse rate, temperature, appetite, vigour, and other factors in the general condition of the patient, including her own desires and sensations; all these must be taken account of before a decision is arrived at. In a succeeding article it will be indicated what is their relative importance.

